# Factors related to loss to follow-up among people living with HIV: a systematic review

**DOI:** 10.1590/S1678-9946202567053

**Published:** 2025-08-18

**Authors:** Zeca Manuel Salimo, Vivian Iida Avelino-Silva, Elizangela Farias da Silva, Yury Oliveira Chaves, Michele Rocha de Araujo El Kadri, Paulo Afonso Nogueira, Adele Schwartz Benzaken

**Affiliations:** 1 Universidade do Estado do Amazonas, Programa de Pós-Graduação em Medicina Tropical, Manaus, Amazonas, Brazil; 2 Universidade Lúrio, Faculdade de Ciências de Saúde, Nampula, Nampula, Mozambique; 3 Universidade de São Paulo, Faculdade de Medicina, Departamento de Doenças Infecciosas e Parasitárias, São Paulo, São Paulo, Brazil; 4 Universidade do Estado do Amazonas, Centro de Estudos Superiores de Tabatinga, Tabatinga, Amazonas, Brazil; 5 Fundação Oswaldo Cruz, Instituto Leônidas e Maria Deane, Programa de Pós-Graduação em Biologia da Relação Patógeno-Hospedeiro, Manaus, Amazonas, Brazil; 6 Fundação Oswaldo Cruz, Instituto Leônidas e Maria Deane, Manaus, Amazonas, Brazil; 7 Universidade Federal do Amazonas, Programa de Pós-Graduação em Imunologia Básica e Aplicada, Manaus, Amazonas, Brazil; 8 AIDS Healthcare Foundation, Los Angeles, California, USA

**Keywords:** HIV, Acquired immunodeficiency syndrome, Follow-up studies, Lost to follow-up, Risk factors

## Abstract

Loss to follow-up (LTFU) among people living with HIV (PLHIV) is a concerning reality in various healthcare services and can occur at any stage of HIV care. LTFU can lead to a decline in overall health and quality of life for PLHIV; moreover, antiretroviral therapy (ART) interruption increase the risk of HIV sexual transmission. This systematic review investigated factors related to LTFU among PLHIV. The review included sources from PubMed, Cochrane Library, Embase, and others. We included observational studies published in English, Spanish, or Portuguese, from January 1, 2004, to December 31, 2024. We identified 36 studies from 20 countries in North and South America, Europe, Asia, and Africa. The studies included 69,789 PLHIV, of whom 22% were classified as LTFU. The time frame used to define LTFU varied across studies, ranging from 14 to 365 days. The most frequently reported factors associated with LTFU were younger age, low educational level, financial instability, illicit drug use, stigma, absence of family and social support, and ART side effects. Other relevant factors included long commuting time to healthcare facilities, long waiting time at health units, and issues with provider-patient relationships. Multiple factors may contribute to LTFU among PLHIV in complex and context-dependent ways. To address this issue, healthcare services must develop a comprehensive understanding of the communities they assist, recognizing distinct subgroups and their specific needs. Public health policies should be implemented to promote continuous care for PLHIV, including early diagnosis, multidisciplinary assessment, and social support.

## INTRODUCTION

Retention in follow-up is essential within clinical care for people living with HIV (PLHIV)^1^. The UNAIDS 95-95-95, aimed at ending the HIV/AIDS epidemic by 2030^2^, set the goals for 95% of PLHIV being aware of their serological status; with 95% of those diagnosed receiving antiretroviral treatment (ART), and with 95% of those on ART achieve an undetectable viral load^2^. Achieving these targets requires a thorough understanding of and substantial investment in strategies to promote patient adherence to uninterrupted care^3^. From a public health perspective, retention in care is also key for controlling the HIV epidemic since sexual transmission of HIV does not occur from PLHIV under ART with an undetectable viral load^4^. However, loss to follow-up (LTFU) remains a common occurrence among PLHIV in healthcare services worldwide^5-8^. For instance, Zago *et al*.^9^ reported a 25% LTFU rate among PLHIV enrolled in a specialized HIV service in Vitoria city, Brazil. Similar findings were reported in the United States^8,10^, Mozambique^11-13^, Uganda^14,15^, and Zambia^16,17^.

Adherence is crucial for the effectiveness of HIV treatment^3,18^, involving dynamic and multifactorial processes encompassing physical, psychological, social, cultural, and behavioral aspects. It requires collaborative decision-making and shared responsibility between PLHIV, healthcare providers, and social support networks^18^. Although retention in care is relevant for managing any chronic condition, HIV-associated stigma may uniquely influence the risk of LTFU among PLHIV^19^. Such stigma may occur within healthcare services^20^, creating direct barriers continued care. Stigma may also originate from family, friends, coworkers, and other social interactions. In a study including 1,784 PLHIV in Brazil, 64% had suffered some form of stigma or discrimination due to their HIV status, and 46% reported being significantly affected by it^21^. Internalized stigma may also negatively influence treatment adherence^22^. Stigma may also affect the extent to which individuals tolerate the side effects of antiretrovirals without discontinuing treatment. For instance, stigmatizing side effects associated with first-generation antiretrovirals, such as lipodystrophy, may impact self-esteem and treatment adherence^23^.

LTFU is a complex issue with multiple determinants, including social and behavioral factors. It may be defined according to different criteria, including failure to pick up antiretroviral medications from the pharmacy or healthcare unit withing the expected timeframe; missing a scheduled appointment; or failing to attend follow-up visits within a specified period^24,25^. Some studies suggest increasing trends in LTFU among PLHIV^11,18^. Despite the significant challenges in ensuring retention in care and sustained ART adherence, LTFU-associated factors remain not fully understood.

This systematic review aims to summarize the available evidence on factors associated with LTFU among PLHIV. Understanding the determinants and context of LTFU in this population could provide valuable insights for developing interventions and strategies to address this pressing challenge.

## MATERIALS AND METHODS

### Search strategy and selection criteria

This systematic review followed the Preferred Reporting Items for Systematic Review and Meta-Analysis (PRISMA) guidelines^26^. The initial research included the databases PubMed, Cochrane Library, and Embase. Additional articles, retrieved from citations of included articles, were also selected. We selected studies published from January 1, 2004, to December 31, 2024, reporting factors associated with LTFU among adult PLHIV. The following search terms were used: “loss to follow-up,” “discontinuation,” “drop out,” “out of care,” “abandonment,” “stopping,” “interruption,” and “unstable patients” (Supplementary Table S1). All articles were imported into EndNote reference management software, version X9 (Clarivate), whereby duplicates were excluded using the “Find Duplicates” function, as described by Bramer^27^. Two investigators (ZMS and EF da S) independently screened titles and abstracts to identify studies relevant to the research objectives. A third investigator (PAN) resolved any discrepancies. The screening process included peer-reviewed original studies published in English, Portuguese, and Spanish, with LTFU timeframes defined according to the Brazilian Ministry of Health guidelines. We included observational studies (cross-sectional, case-control, and cohort studies) that enrolled PLHIV aged 18 years and older, regardless of sex.

Full texts for studies selected based on title and abstract were reviewed by four independent reviewers (ZMS, EF da S, PAN, and ASB) to extract data on factors associated with LTFU among PLHIV. Title, journal, URL or DOI address, study location, year of publication, data source, study objectives, study design, sample size, total number of participants classified as LTFU stratified by sex, duration of LTFU, and factors associated with LTFU identified in each study were extracted and organized in a standardized form (Supplementary Table S2). Studies were then categorized according to study design, including cross-sectional, retrospective cohort, prospective cohort, and case-control. Our search did not identify clinical trials meeting the selection criteria. Studies with outcomes unrelated to the proposed topic or with a design outside our inclusion criteria were excluded.

### Risk of bias assessment

All studies meeting the inclusion criteria underwent a risk of bias analysis conducted independently by two authors (ZMS and EFS) using the Cochrane Collaboration “Risk of Bias” tool^28^. Six criteria were evaluated across four types of bias: selection bias, performance and detection bias, attrition bias, and reporting bias (Supplementary Table S3).

### Ethical considerations

This study is based exclusively on published literature and did not require approval from an ethics review board.

### Data analysis

The SPSS statistical software (version 21.0, SPSS Inc., Chicago, IL, USA) was used for all analysis. From the extracted data, we calculated the percentage of LTFU stratified by sex for each study using the following formula: n/N*100%; *n*1/*N*1*100%; Σ*F* + *M*, in which: F: female; M: male; T: total; %: percentage; n: number of partial sample included in the study stratified by sex; N: total sample included in the study; n1: number of participants LTFU stratified by sex; N1: total number of participants LTFU among PLHIV.

Although some studies identified similar factors associated with LTFU, we observed substantial heterogeneity in terms of design and definitions, preventing us to perform a meta-analysis. Therefore, we opted for a narrative review. Factors associated with LTFU reported in the studies were categorized into three subgroups: sociodemographic factors, clinical factors, and service-related factors. We used GraphPad Prism version 9.02 to develop graphical representations of our findings.

## RESULTS

### Study selection

**Figure 1:**
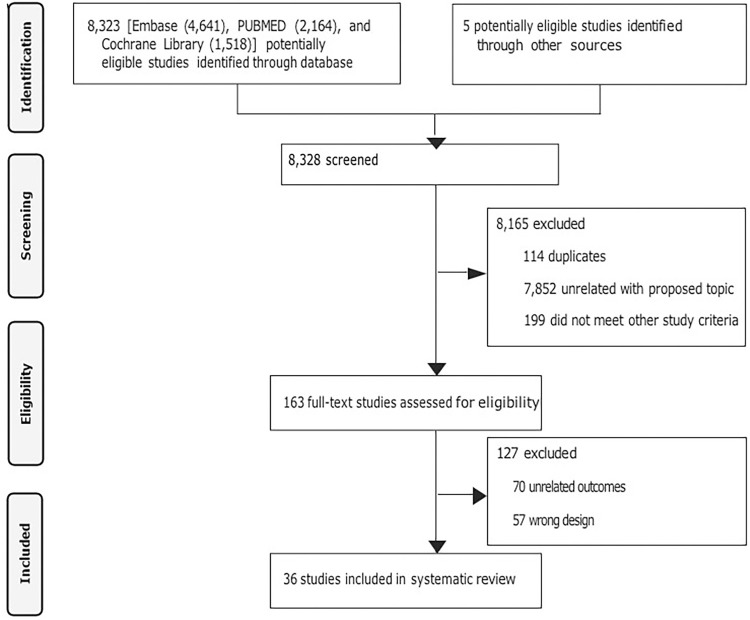
Flowchart of the study selection process.

A total of 8,328 citations were identified via database searches and other sources, including publications retrieved from citations within the included articles. Of these, 8,165 were excluded after title and abstract screening. These A total of 114 duplicates, 7,852 studies unrelated to the topic, and 199 studies that did not meet at least one inclusion criteria were excluded. The remaining 163 studies were selected for full-text review, of which 127 were excluded. Ultimately, 36 articles were included in this systematic review (Figure 1).

### Quality assessment and risk of bias

Studies selected for this analysis generally exhibited poor quality in terms of study design, with a moderate risk of bias (Supplementary Table S3).

### Characteristics of studies included in the systematic review

**Figure 2:**
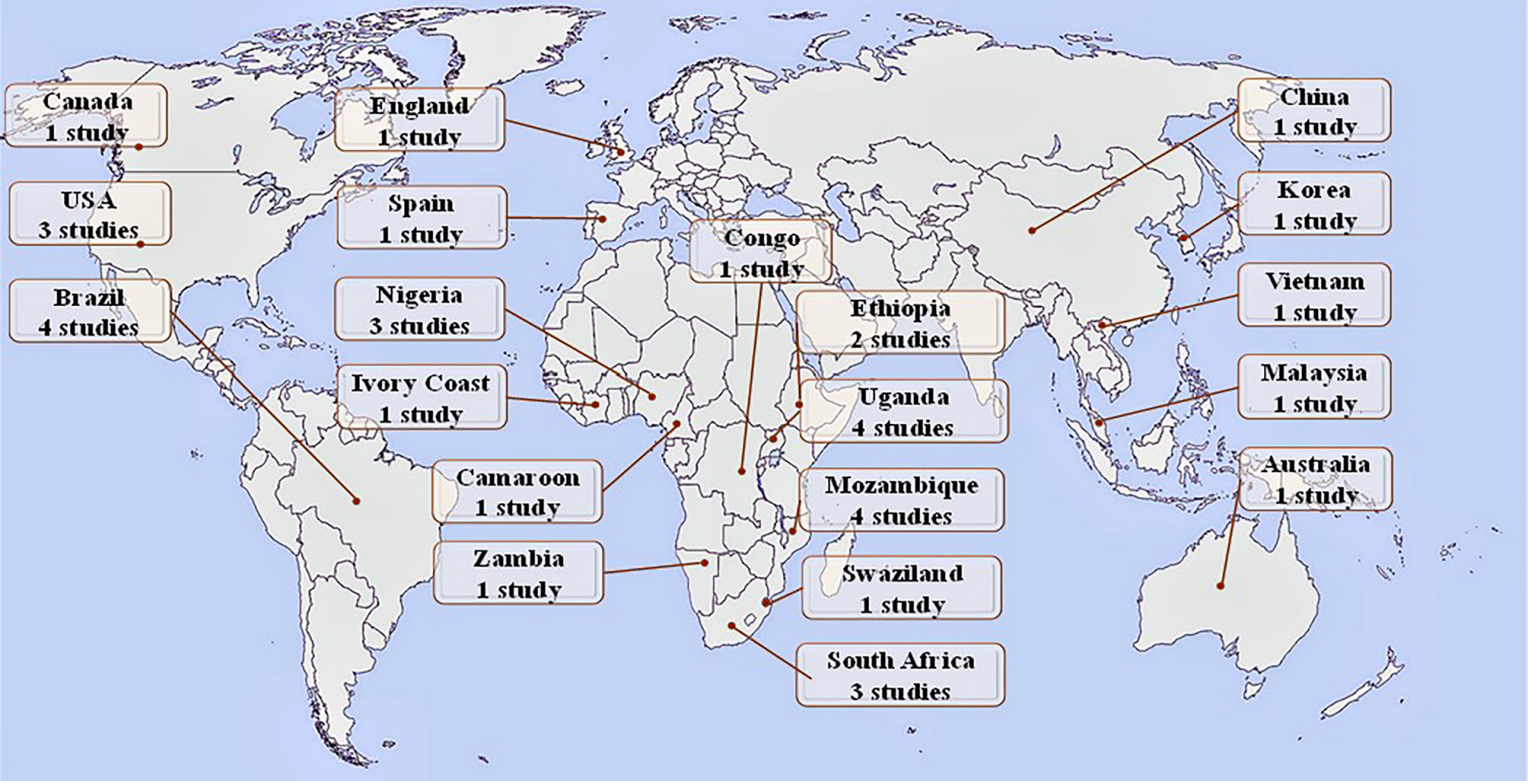
Number of eligible studies analyzed per country.

A total of 36 primary studies from 20 different countries were included (Figure 2). The study designs were as follows: 12 cross-sectional studies, 18 retrospective cohort studies, five prospective cohort studies, and one case-control study (Table 1). These articles comprehensively addressed the topic by evaluating the characteristics of PLHIV who experienced LTFU, describing the participants and providing data on factors leading to LTFU among adult PLHIV. Some studies included a qualitative component, primarily comprising structured interviews.

**Table 1:** Characteristics of studies included in the analysis.

Articles	Number of participants			Participants LTFU			LTFU time (days)	Age (years)		Study duration (Months)	Study design	Location
	F n (%)	M n (%)	T N	F n1 (%)	M n1 (%)	T N1 (%)		Mean	Median			
Muga *et al*.^37^	10 (11.63)	76 (88.37)	86	7 (70.0)	22 (28.95)	29 (33.72)	>30	32	-	35	Cross-sectional cohort study	Barcelona, Spain
Cardoso and Arruda^7^	16 (50.0)	16 (50.0)	32	UN	UN	16 (50.0)	>90	-	34.5 (30 -39)	UN	Retrospective cohort study	Rio de Janeiro, Brazil
Yuan *et al*.^8^	479 (14.0)	2935 (86.0)	3414	237 (49.4)	1104 (37.6)	1341 (39.3)	≥14	≥18	-	78	Retrospective cohort study	Vienna, USA
Robison *et al*.^38^	186 (25.21)	552 (74.79)	738	18 (9.68)	77 (13.95)	95 (12.87)	≥365	≥18	-	102	Retrospective cohort study	Birmingham, England
Asad *et al*.^48^	1070 (29.0)	2584 (71.0)	3654	59 (5.52)	130 (5.04)	183 (4.92)	≥90	≥18	-	78	Cross-sectional cohort study	Tennessee, USA
Duff *et al*.^41^	45 (100.0)	0	45	14 (31.11)	0	14 (31.11)	>90	≥18	-	36	Retrospective cohort study	Western Uganda
Miller *et al*.^42^	396 (75.0)	129 (25.0)	528	UN	UN	17 (3.22)	>30	31	-	48	Prospective cohort study	South Africa
Kranzer *et al*.^46^	752 (65.2)	402 (34.8)	1154	172 (14.90)	115 (9.96)	287 (24.87)	>30	-	31.9 (27.3-37.50	70	Retrospective cohort study	Cape Town, South Africa
Geng *et al*.^6^	2213 (61.0)	1415 (39.0)	3628	59 (2.31)	20 (1.41)	79 (2.18)	≥183	-	35 (30-42)	48	Prospective cohort study	Mbarara, Uganda
Groh *et al*.^12^	76 (45.68)	88 (54.32)	162	UN	UN	162 (100.0)	>60	30	-	12	Cross-sectional cohort study	Zambezia, Mozambique
Schilkowsky *et al*.^35^	340 (36.0)	605 (64.0)	945	51 (15.0)	104 (17.19)	155 (16.40)	>90	35	-	115	Prospective cohort study	Rio de Janeiro, Brazil
Musheke *et al.* ^16^	17 (68.0)	8 (32.0)	25	17 (68.0)	8 (32.0)	25 (100.0)	>30	≥18	-	7	Prospective cohort study	Lusaka, Zambia
Zago *et al*.^9^	87 (46.3)	101 (53.7)	250	22 (25.28)	40 (39.60)	62 (24.8)	>90	-	39.5 (33-48)	12	Case-control study	Vitoria, Brazil
Tran *et al*.^39^	862 (25.0)	2587 (75.0)	3449	104 (12.06)	426 (16.47)	530 (15.37)	>90	≥18	-	48	Prospective cohort study	Vietnam
Hughes *et al*.^10^	1212 (30.87)	2714 (69.13)	3926	101 (8.34)	117 (4.31)	218 (5.55)	>90	≥18	-	9	Cross-sectional cohort study	USA
McManus *et al*.^49^	205 (6.0)	3208 (94.0)	3413	108 (52.68)	2241 (69.85)	2349 (68.82)	≥365	42.3	-	96	Cross-sectional cohort study	Australia
Vuylsteke *et al*.^29^	376 (90.82)	38 (9.18)	414	UN	UN	195 (47.0)	≥90	32	-	36	Retrospective cohort study	Côte d’Ivoire
Samji *et al*.^47^	1451 (19.0)	6182 (81.0)	7633	573 (39.49)	1287 (20.81)	1860 (24.37)	>90	-	38 (32-44)	132	Cross-sectional cohort study	Canada
Agbaji *et al*.^30^	8005 (66.6)	4008 (33.4)	12013	2126 (26.6)	1236 (30.8)	3362 (27.98)	>360	-	34 (29-41)	12	Retrospective cohort study	Nigeria
Marega *et al*.^11^	76 (51.0)	73 (49.0)	149	77 (51.0)	73 (49.0)	149 (100.0)	>60	35	-	12	Cross-sectional cohort study	Chiure, Mozambique
Rodrigues and Maksud^44^	3 (37.5)	5 (62.5)	8	3 (37.5)	5 (62.5)	8 (100.0)	>90	≥18	-	6	Retrospective cohort study	Rio de Janeiro, Brazil
Pires *et al*.^54^	206 (69.8)	89 (30.2)	295	54 (26.2)	32 (36.0)	86 (29.2)	>60	-	40 (18-62)	12	Cross-sectional cohort	Nampula, Mozambique
Akilimali *et al*.^55^	479 (68.8)	238 (31.2)	717	48 (10.02)	38 (15.97)	86 (12.0)	≥90	38.2	-	96	Retrospective cohort study	Goma, Congo
Shabalala *et al*.^36^	84 (58)	61 (42)	52	UN	UN	11 (21.15)	>30	≥18	-	34	Retrospective cohort study	Swaziland
Ibiloye *et al*.^31^	549 (77.3)	161 (22.7)	710	UN	UN	166 (23.4)	>90	-	30 (24-35)	7	Retrospective cohort study	Nasarawa, Nigeria
Seifu *et al*.^50^	850 (58.0)	589 (42.0)	1439	106 (12.47)	107 (18.17)	213 (14.8)	≥90	33.5	-	12	Retrospective cohort study	Jigjiga, Ethiopia
Opio *et al*.^15^	391 (60.5)	255 (39.5)	646	35 (8.95)	12 (4.70)	216 (33.4)	>90	≥18	-	24	Retrospective cohort study	Wakiso, Uganda
Assemie *et al*.^53^	180 (54.5)	150 (45.5)	330	UN	UN	73 (22.12)	>90	33.3	-	12	Cross-sectional cohort study	Pawi, Ethiopia
Balogun *et al*.^52^	4065 (66.6)	2043 (33.4)	6108	168 (62.7)	100 (37.3)	268 (4.4)	>180	-	39 (34-45)	6	Cross-sectional cohort	Lagos, Nigeria
Kiwanuka *et al*.^32^	4857 (64.3)	2696 (35.7)	7553	746 (15.36)	434 (16.09)	1180 (15.62)	>90	≥18	-	55	Retrospective cohort study	Musaka, Uganda
Palombi and Moda^33^	UN	UN	82	UN	UN	82 (100.0)	>60	≥18	-	3	Retrospective cohort study	Mangunde, Mozambique
Nsoh *et al*.^43^	204 (75.3)	67 (24.7)	271	204 (100.0)	67 (100.0)	271 (100.0)	>30	33	-	12	Cross-sectional cohort study	Nkolndongo, Cameroon
Siti-Azrin *et al*.^56^	38 (13.8)	238 (86.2)	276	12 (19.0)	51 (81.0)	63 (22.82)	>90	≥18	-	19	Retrospective cohort study	Selangor, Malaysia
Ma *et al*.^34^	124 (4.95)	2382 (95.05)	2506	24 (19.35)	288 (12.09)	312 (12.45)	>30	-	31 (26-40)	192	Retrospective cohort study	Jinan, China
Seong *et al*.^59^	223 (7.03)	2949 (92.97	3172	91 (40.80)	1315 (44.59)	1406 (44.33)	≥365	-	35 (27-44)	120	Cross-sectional cohort study	Korea
Modipane *et al*.^51^	18 (90.0)	2 (20.0)	20	18 (100.0)	2 (100.0)	20 (100.0)	>90	≥18	-	3	Retrospective cohort study	Sekhukhune, South Africa
Total	30143 (43.19)	39646 (56.81)	69789	6138 (20.36)	9451 (23.84)	15589 (22.33)						

F = female; M = male; T = total; % = percentage; n = number of partial sample included in the study stratified by gender; N = total sample included in the study; n1 = number of participants who LTFU stratified by gender; N1 = total number of participants who LTFU among PLHIV; UN = undetermined; > = greater than; ≥ = greater or equal.

Table 1 summarizes the characteristics of all studies included in this systematic review, including number of participants, number of PLHIV who were LTFU stratified by sex, duration of LTFU, study design, study period and location. Regarding participants’ characteristics, the most common age range was 18 to 44 years of age, with a predominance of males (57%) (Table 1). Common socioeconomic issues included low education^11,29-34^, unemployment^6,16,31,34-36^, illicit drugs use^9,35,37-40^, and economic difficulties^6,10-12,16,32,38,41-43^.

The number of participants varied across studies, ranging from as few as eight PLHIV from a study conducted in Rio de Janeiro, Brazil^44^, to as many as 12,013 participants in a study conducted in Nigeria^30^. Our review included a total of 69,789 PLHIV, of whom 15,589 (22.33%) were LTFU. Among the latter, 61% were male; the percentage of women living with HIV who were subsequently LTFU was 20%, compared to 24% among men. The criteria for defining LTFU varied by country but generally involved not collecting ART from the health unit’s pharmacy for more than 90 days.

### Factors related to LTFU among PLHIV

Structured interviews and medical records analysis were the most common methods to identify factors associated with LTFU^9,11,30,33,35,41,44-46^. For better organization, we categorized these factors into three subgroups: sociodemographic, clinical, and service-related factors.

### Sociodemographic factors related to LTFU among PLHIV

**Figure 3:**
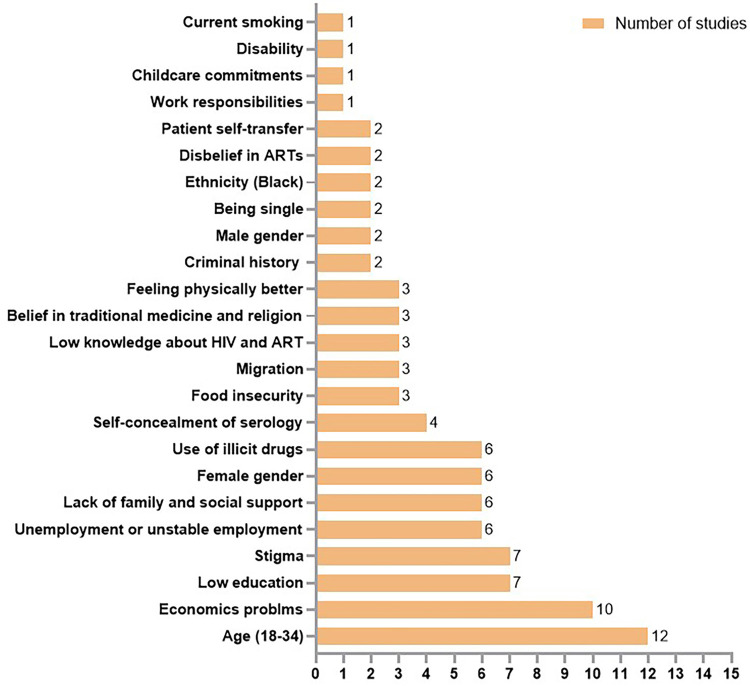
Sociodemographic factors related to LTFU among PLHIV.

Risk factors identified in this category included: sex, younger age (18–34 years), single marital status, low education or illiteracy, economic difficulties, unemployment or unstable employment, work obligations, childcare responsibilities, food insecurity, lack of family or social support, stigma, self-concealment of HIV status, limited knowledge about HIV and ART, and disbelief in ART. Additional relevant factors included belief in traditional medicine and religion, perceived physical improvement, disability, patient self-transfer, Black skin color, current smoker, use of illicit drugs, and criminal history (Figure 3).

Twelve studies found that younger PLHIV were more likely to experience LTFU, specifically those aged 18 to 34 years^8,10,15,16,29,30,35,38,39,47-49^. Schilkowsky *et al*.^35^ observed that the risk of LTFU was higher among PLHIV aged 18 to 29 years, with a reduction of approximately 5.0% for each additional year of age. A similar study in Lusaka, Zambia, identified that PLHIV aged 25 to 34 years were at higher risk of LTFU^16^. In Canada, the adjusted hazard ratio (aHR) for LTFU among younger PLHIV was 1.26 (aHR = 1.26; 95% CI: 1.17–1.81)^47^. In Nigeria, one study found that the risk of LTFU among PLHIV decreases with increasing age (aHR = 0.99; 95% CI: 0.98–0.99)^30^. These findings were consistent across multiple studies^8,10,15,16,29,30,35,38,39,47-49^, showing that younger age is a significant predictor of LTFU among PLHIV.

Six studies linked LTFU among PLHIV with the female sex^10,16,37,39,47,48^. Muga *et al*.^37^ reported that approximately 70% of female PLHIV in Spain were LTFU during clinical follow-up , compared to 28.95% of males, indicating a 7.9-fold risk among women (RR = 7.9; 95% CI: 0.0–69.5). Similar findings were reported in Zambia^16^ and Canada^47^, where LTFU among women was 68.0% and 34.49%, respectively, compared to 32.0% and 20.81% among men. Other studies^10,39,48^ reported similar trends. However, contrasting results were observed in South Africa, in which LTFU was more frequent among men (16.6%) than women (11.1%)^46^. Similar results were reported in Ethiopia^50^.

Two studies reported associations between being single and increased risk of LTFU among PLHIV^30,35^. Schilkowsky *et al*.^35^ found that single participants had a 1.5 times higher risk of LTFU compared to their married counterparts (aHR = 1.502; 95% CI: 1.007–2.240). A similar study conducted in Nigeria reported a risk ratio of 1.24 for single PLHIV (aHR = 1.24; 95% CI: 1.12–1.38)^30^.

Two studies associated Black ethnicity with higher LTFU rates among PLHIV^8,48^. In a U.S.-based study, Black PLHIV had higher risk of LTFU, with a relative risk (RR) of 1.28 (95% CI: 1.13–1.45) compared to White participants^8^. Another U.S. study reported a similar finding (RR = 1.46; 1.08–1.99; P = 0.015)^48^.

Three studies found that perceived physical improvement was associated with LTFU^51-53^. In Nigeria, 6.7% of PLHIV were lost to follow-up because they felt healthy^52^. In Ethiopia, 32.9% of PLHIV stopped taking ART because they believed they had been cured^53^. Similar observations were made in South Africa^51^. Conversely, PLHIV with physical or mental disabilities were also at high risk of LTFU^48^.

Seven studies associated low educational levels or illiteracy to higher rates of LTFU^11,29-34^. In Nigeria, PLHIV with low education levels had an increased risk of LFTU (aHR = 1.24; 95%CI: 1.12–1.40)^30^. Similar findings were observed in other studies conducted in Nigeria^31^ Mozambique^11^, Uganda^32^, Côte d’Ivoire^29^, and China^34^.

Ten studies associated LTFU among PLHIV with economic difficulties^6,10-12,16,31,32,38,41,42^. In Western Uganda, 93% of PLHIV who were LTFU reported low economic conditions, often related with difficulties in accessing transportation to healthcare facilities^41^. Similar findings were reported in South Africa^42^ and Zambia^16^. In Mozambique, 162 PLHIV were classified as LTFU due to financial difficulties related to food and transportation^12^; this was observed in another report from Mozambique^11^. In Uganda, PLHIV without a personal telephone number for follow-up contact had a higher risk of LTFU (aHR = 1.69; 95%CI: 1.50–1.91)^32^. Other studies reported similar results^6,10,31,38^.

Six studies linked LTFU among PLHIV to unemployment or unstable employment^6,16,31,34-36^. In a Brazilian study, LTFU risk was significantly higher among PLHIV with unstable jobs^35^; similar findings were reported in Eswatini^36^. Musheke *et al*.^16^ reported that fear of job loss contributed to LTFU among PLHIV in Zambia. In Nigeria, unemployed individuals had an increased risk of LTFU (aHR = 1.8; 95%CI: 1.2–2.6)^31^. Work responsibilities and childcare commitments were also associated with LTFU^6^.

Three studies associated LTFU among PLHIV with food insecurity^12,33,54^. Food insecurity was the most frequently reported factor among PLHIV who were LTFU, affecting approximately 42.9% of cases^33^. Similar findings were reported in Mozambique^12,54^.

Migration or nomadism was also linked to LTFU among PLHIV^33,36,51^. In Eswatini, patient mobility in search of better living conditions was the key contributor to LTFU^36^. Similarly, Moda and Palombi^33^ reported that 11.4% of LTFU cases among PLHIV in Chibabava, Mozambique, were influenced by nomadism. Additionally, self-transfer of patients was associated with a higher risk of LTFU in South Africa^51^.

Six studies found that a lack of family and social support contributed to LTFU^6,7,10,11,16,36^. In a Brazilian study, emotional distress and family issues were linked to LTFU^7^. In Zambia, fear of losing family support was a significant contributor to LTFU^16^. Marega *et al*.^11^ identified that the absence of a confidant was associated with LTFU among PLHIV in Mozambique. In Eswatini, the lack of a social support was also related to LTFU^36^, and similar results were reported in the United States^10^. In Uganda, PLHIV discontinued care after being advised by their families not to return to the healthcare unit (aHR = 4.2; 95%CI: 0.5–14.0)^15^.

Seven studies reported associations between stigma and LTFU^11,15,16,33,41,43,54^. In a study conducted in Uganda, 58% of LTFU cases were attributed to stigma related to HIV status^41^. Similar findings were observed in Mozambique^11,54^. Musheke *et al*.^16^ reported that fear of anticipated stigma was a significant contributor to LTFU in Zambia. Nsoh *et al*.^43^ identified stigma as the most prominent predictor of treatment interruption (47.5%) among PLHIV in Cameroon; similar results were reported in Uganda^15^.

Four studies showed that LTFU among PLHIV was related to non-disclosure of serological status^16,41,50,55^. In Uganda, fear of having their serological status discovered—along with associated stigma—were significant reasons for LTFU, affecting 58% of participants^16^. Similar observations were reported in Zambia^41^. In The Republic of the Congo, PLHIV who did not share their HIV status had a significantly higher risk of LTFU (aHR = 2.28; 95%CI: 1.46–2.29)^55^. Likewise, in Ethiopia, non-disclosure was related to higher risk of LTFU (aHR = 2.8; 95%CI: 2.22–5.23)^50^.

Lack of knowledge about HIV and ART was related to LTFU among PLHIV in a study performed in Zambia^16^. Contributing factors included disbelief in the effectiveness of ART to improve quality of life, as well as a preference for traditional medicine and religious beliefs in healing^16^. Likewise, Duff *et al*.^41^ reported that 60% of PLHIV who abandoned ART in Uganda were unaware of highly active antiretroviral therapy (HAART).

Six studies identified that illicit drug use contributed to LTFU among PLHIV^9,10,35,37,38,47,49^. In a Brazilian study, drug users had 2.3 times higher odds of experiencing LTFU compared to non-users (adjusted odds ratio [aOR] = 2.3; 95%CI: 1.03–5.07)^9^. In Canada, the risk was also elevated among drug users (aHR = 1.46; 95% CI: 1.09–1.89)^36^, with similar observations reported in Spain^37^. Schilkowsky *et al*.^35^, in a study from Rio de Janeiro, Brazil, found that LTFU was associated both with illicit drug use and with having a criminal record. Similar findings were reported in England^38^, the United States^10^, and Australia^49^. Moreover, a U.S. study found that current smoking was also related to higher risk of LTFU (RR = 1.33; 95% CI: 1.18–1.50)^8^.

### Clinical factors related to LTFU among PLHIV

**Figure 4:**
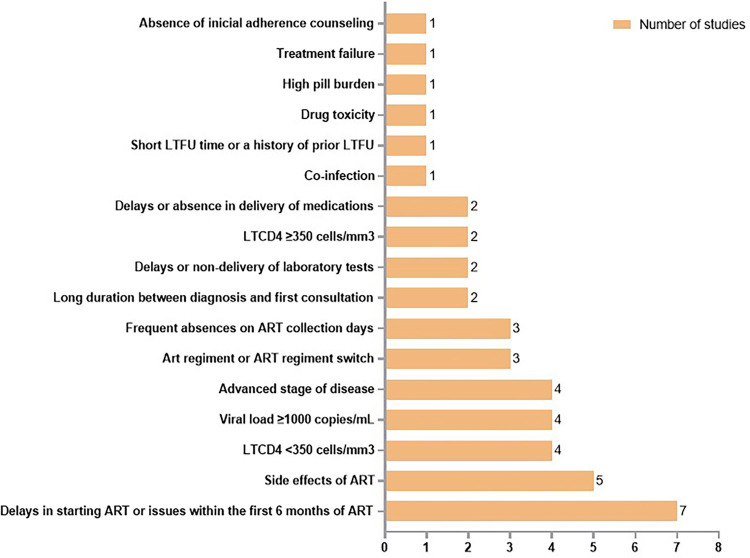
Clinical factors related to LTFU among PLHIV.

Several clinical factors have been associated with LTFU among PLHIV. These include long delay between diagnosis and the first appointment; delays or failure in delivering laboratory test results; low CD4+ T cell counts; viral load >1,000 copies/mL; advanced disease stage; absence of initial adherence counseling; delays or interruptions in medication delivery; issues with ART adherence; and ART regimen modification. Additional contributing factors include delayed initiation of ART; adherence issues within the first 6 months of ART; prior episodes of LTFU; missing visits in ART dispensation days; high pill burden; ART-related side effects or drug toxicity; and treatment failure (Figure 4).

The time interval between HIV diagnosis and the first medical appointment has been linked to LTFU among PLHIV^35^. A Brazilian study found a statistically significant difference (p = 0.0051) in this lag time between diagnosis and first medical consultation among PLHIV who were lost to follow-up (average delay of 1 year and 4 months for the LTFU group, compared to 7.5 months for those in regular follow-up^35^. These findings suggest that a longer interval between diagnosis and the first appointment is related to higher likelihood of LTFU, similar to a finding reported in the United States^10^.

Two studies found a relationship between delays or non-delivery of laboratory test results and LTFU among PLHIV^16,44^. In Zambia, one study found that some participants reported that they discontinued care due to not receiving test results^16^. Similarly, a study from Brazil identified that delays in delivering laboratory results contributed to LTFU^44^.

Four studies associated LTFU among PLHIV with CD4+ T cell counts <350 cells/mm^3^, viral load >1,000 copies/mL, and advanced disease stage^9-11,32,41^. A Brazilian study found higher odds of LTFU among PLHIV with CD4+ counts <200 cells/mm^3^ (aOR = 1.5; 95%CI: 1.03–2.10) and among those with viral load >1,000 copies/mL (aOR = 2.0; 95%CI: 1.34–3.09)^9^. In Uganda, Kiwanuka *et al*.^32^ reported an increased risk of ART non-adherence among PLHIV with CD4+ cell counts of 200-350 μ/ml (aHR = 1.21; 95%CI: 1.01–1.45) and among those at WHO clinical stage 3 or 4 at baseline (aHR = 1.35; 95%CI: 1.10–1.65). Similar findings were reported in Uganda^41^, Mozambique^11^, and the United States^10^. Conversely, two studies conducted in Canada^47^ and South Africa^46^ also reported LTFU among PLHIV with CD4+ counts ≥350 cells/mm^3^. Additionally, a study from Malaysia found that tuberculosis co-infection was strongly related to LTFU (aOR = 2.0; 95%CI: 1.1–3.7, p = 0.025)^56^.

Two studies reported that LTFU among PLHIV was associated with delays in the delivery of medications^16,44^. In Zambia, participants indicated that abandonment of treatment was driven by unavailability of antiretroviral medications at their health unit, leading to frustration and fatigue due to prolonged waiting times^16^. Similarly, Rodrigues and Maksud^44^ reported that delays in medication delivery at scheduled monthly visits contributed significantly to LTFU among PLHIV in Rio de Janeiro, Brazil. Additionally, the lack of initial adherence counseling was also related to LTFU among PLHIV in Côte d’Ivoire^29^.

Seven studies reported that delayed ART initiation was associated with a higher risk of LTFU among PLHIV^8,29,32,34,46,47,49^. In Uganda, patients who initiated ART more than seven days after diagnosis had a higher risk of LTFU (aHR = 1.69; 95%CI: 1.50–1.91)^32^. Similar findings were reported in studies from Canada^47^ and South Africa^46^. In China, Ma *et al*.^34^ found that delayed ART initiation was related to a higher risk of LTFU (aHR = 1.43; 95%CI: 1.10–1.85). Other studies also reported similar results^8,29,49^.

Three studies associated the type of ART regimen or a regimen switch with LTFU among PLHIV^34,47,56^. In Canada, patients who initiated treatment with zidovudine had a 2.47-fold higher risk of LTFU compared to those receiving tenofovir (aHR = 2.47; 95%CI: 1.92–3.20)^47^. A study in China reported that patients starting ART with tenofovir alafenamide fumarate had a higher risk of LTFU compared to those receiving zidovudine (aHR = 5.19; 95%CI: 3.29–8.21)^34^. Interestingly, in Malaysia, a history of ART regimen switch was significantly associated with LTFU (aOR = 5.3; 95%CI: 2.2–13.1; p<0.001)^56^.

The frequency of missed ART dispensation visits was also related to LTFU among PLHIV^30^. In Nigeria, PLHIV with an average adherence rate <95% for medication pickup appointments had significantly higher risk of LTFU (aHR = 2.13; 95%CI 1.9–2.40)^30^. A history of prior or short-term LTFU was also identified as a predictor of subsequent LTFU^49^.

Five studies associated ARV side effects with LTFU among PLHIV^7,12,16,36,53^. In a Brazilian study, ART side effects were the primary reason reported for discontinuing care^7^. Similarly, ART side effects accounted for 8.0% of LFTU cases in rural districts of Mozambique^12^. Similar results were observed in Zambia^16^ and Eswatini^36^. Additional factors contributing to LFTU included high pill burden, drug toxicity, and treatment failure, as reported by a study from the United States^8^.

### Service-related factors related to LTFU among PLHIV

**Figure 5:**
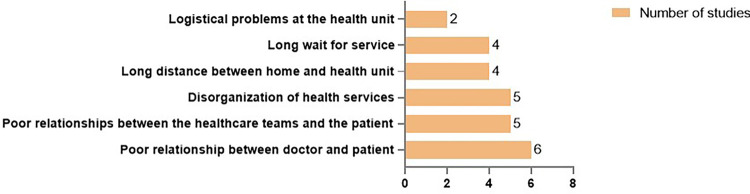
Service-associated factors related to LTFU among PLHIV.

In the risk factors category, LTFU among PLHIV was associated with long distances between patients’ residences and healthcare facilities, logistical issues within health units, and extended waiting times for care. Notably, poor patient-provider relationships were also reported as significant contributors to LTFU (Figure 5).

In a study from Zambia, long distances between patients’ residences and the healthcare unit was related to LTFU among PLHIV, resulting in 25 participants being LTFU^16^. Seven studies identified several service-related factors contributing to LTFU, including logistical issues, disorganization within healthcare services, and long waiting times for care^11,16,33,36,41,42,44^. For instance, a study from South Africa reported that logistical issues—such as inadequate referral and transfer processes, lack of patient advocates, and limited outpatient hours on weekends—exacerbated LTFU^42^. In Zambia, PLHIV reported long wait times, poor quality of care, and fatigue due to lengthy clinical appointments as reasons for LFTU^16^. Similar issues were reported in Mozambique^11,33^, Brazil^44^, Uganda^41^, and Eswatini^36^.

Six studies associated LTFU with poor patient-provider relationships^12,16,36,41,44,55^. In Mozambique, 42% of LTFU were attributed to poor relationships and/or conflicts between healthcare providers and PLHIV^12^. A similar finding was reported in Eswatini^36^. In Zambia, participants cited rudeness from facility staff as a reason for LTFU^16^. Likewise, in a study from Uganda, 32% of LTFU cases were linked to poor relationship between doctors and patients^41^, and similar findings were observed in Brazil^44^.

## DISCUSSION

In this study, we systematically investigated the available evidence regarding factors related to LTFU among PLHIV, based on 36 original observational studies conducted across 20 countries. Our analysis identified three main categories of factors contributing to LTFU: sociodemographic, clinical, and service-related factors.

Although several studies identified similar factors related to LTFU, the underlying motivations are multifactorial, complex, and context-specific. Healthcare services must consider the diverse experiences and the needs of different population groups, each of which faces distinct challenges in how they live, experience illness, and access care^18^. Overlooking these specific characteristics may increase the risk of LTFU, compromising the quality of life of PLHIV and hindering the progress towards achieving the UNAIDS 95-95-95 targets to end the HIV epidemic by 2030^2^.

Several studies in our analysis found that PLHIV aged 18–34 years and those who are single are at higher risk of LTFU^8,10,15,16,29,30,35,38,39,47-49^. This increased risk among the youth may be influenced by factors related to cognitive, sexual, psychological, and physical development^57^. These findings highlight the need to intensify counseling programs on HIV/AIDS and ART within healthcare settings to improve retention rates among young PLHIV.

Our findings also indicate females may be at greater risk of LTFU among PLHIV^10,16,37,39,47,48^; this results, however, are inconsistent across studies^10,39,48^, suggesting that gender-related factors influencing LTFU are contextual. In Zambia, economic dependence and fear of losing a spouse influenced many women to avoid disclosing their HIV status, resulting in LTFU^16^. Empowering women and intensifying human rights awareness could help address this barrier. In Canada, Samji *et al*.^47^ suggested that the higher risk among women could be partially attributed to ART initiation during pregnancy, followed by treatment discontinuation. Healthcare units should closely monitor pregnant women living with HIV, as they may be particularly vulnerable to LTFU. Additionally, women may experience more frequent and severe ART side effects and toxicities compared to men, due to physiological differences and the influence of sex hormones on drug metabolism^47^.

Factors such as low education or illiteracy, migration or nomadism, unemployment or unstable employment, low economic status, and food insecurity are closely linked to poverty. These variables significantly contributed to LTFU among PLHIV in many studies^11,12,16,30,32-36,54,58^. For example, financial constraints limiting access to transportation and nutrition, as well as the need for frequent relocation in search of better living conditions (nomadism), are poverty-related factors associated with LTFU^33^. Public policies that promote employment opportunities and provide financial support for individuals living in poverty could positively impact retention in care for PLHIV.

Discrimination, lack of family and social support, as well as stigma, can significantly discourage PLHIV from continuing in care^7,11,16,33,36,41,54^. Without social and psychological support, patients may lose confidence and consequently self-exclude from care^11^. To address these issues, we suggest implementing public policies that promote social inclusion, provide emotional support, and combat HIV-related stigma. Additionally, educational programs should emphasize themes of love, respect, and inclusion for all individuals, regardless of their HIV status.

Disbelief in ART is another factor contributing to treatment discontinuation. Studies showed that some patients place greater trust in traditional medicine and faith healing^16^. Rather than viewing faith and traditional medicine as obstacles to ART adherence, healthcare providers and peer navigators should be trained to educate patients about the benefits of ART while respecting their beliefs. Use of illicit drugs and a criminal history have also been related to LTFU among PLHIV^9,35,37,47^. Healthcare units should pay special attention to this group, as they often face significant social exclusion that can undermine their confidence and retention in care^35^.

Regarding clinical and laboratory monitoring of PLHIV, timely medical consultations and prompt ART initiation after diagnosis are crucial. Delays between diagnosis and the first appointment have been associated with increased risk of LTFU^35^. It is also essential that laboratory test results are delivered promptly during routine consultations and clearly explained to the patient^10,11,26,52,59^. Explaining the implications of test results and potential consequences of ART abandonment can help mitigate LTFU.

During routine follow-up, patients who frequently miss ART medication pickup appointments should be informed about risks associated with LTFU for PLHIV^30^. A dedicated team should make all efforts to contact or visit these patients to understand and address their needs, concerns, and barriers to care. Previous studies showed that key factors contributing to LTFU include delays in medication delivery, ART regimen issues, treatment adjustments during the first six months of therapy, delayed ART initiation, high pill burden, drug toxicity, treatment failure, and side effects^7,12,16,30,32,34,36,46^. When ART regimen modifications occur, patients should be carefully informed about the reasons for change, potential side effects, and the expected duration of side effects^18^.

Healthcare services are also crucial in preventing LTFU among PLHIV. Distance between a patient’s residence and the healthcare unit can affect LTFU, particularly for economically disadvantaged patients who struggle with transportation costs. Several studies have identified this as a key factor affecting retention in care^11,16^. Thus, health programs should consider restructuring and decentralizing HIV services, including ART distribution sites. Logistical challenges, disorganized services, and long waiting times have also been associated with increased risk of LTFU^11,16,33,36,41,44^. Finally, poor patient-provider relationships have been identified as significant factors related to LTFU^12,16,36,41,44^. HIV services should implement training programs for healthcare providers that emphasize not only technical aspects but also empathy, respect, and human rights.

### Limitations

Although some studies identified similar factors associated with LTFU, we observed substantial heterogeneity in study design and definitions, precluding our ability to proceed with a meta-analysis. Therefore, we opted for a narrative review approach.

## CONCLUSION

LTFU among PLHIV can occur at any stage of clinical care. Sociodemographic, clinical, and health service-related factors may contribute to this outcome. Common sociodemographic factors include young age, low education, financial difficulties, illicit drugs use, stigma, and lack of family and social support. Clinical and service-related contributors include delayed ART initiation, medication side effects, long distances to healthcare facilities, and long waiting times. These factors disrupt treatment continuity, hindering the health and quality of life of PLHIV and undermining efforts to control the HIV epidemic. Appropriate measures to ensure ART adherence should be developed considering diverse community, cultural, and environmental contexts. Healthcare services and providers must understand the specific needs and challenges of the populations they serve. Additionally, public and healthcare policies should be developed and implemented to ensure continuous care for PLHIV, from diagnosis to retention in care.

## Appendix 1

Supplementary Material available from: https://doi.org/10.48331/scielodata.MCFLHW

## References

[1] Leon C, Koosed T, Philibert B, Raposo C, Benzaken AS (2019). HIV/AIDS health services in Manaus, Brazil: patient perception of quality and its influence on adherence to antiretroviral treatment. BMC Health Serv Res.

[2] UNAIDS Global HIV & AIDS statistics: fact sheet.

[3] Polejack L, Machado AC, Santos CS, Guambe AJ (2020). Desafios para a adesão ao TARV na perspectiva dos profissionais do Sistema de Saúde de Moçambique. Psicol Teor Pesq.

[4] Broyles LN, Luo R, Boeras D, Vojnov L (2023). The risk of sexual transmission of HIV in individuals with low-level HIV viraemia: a systematic review. Lancet.

[5] Weigel R, Hochgesang M, Brinkhof MW, Hosseinipour MC, Boxshall M, Mhango E (2011). Outcomes and associated risk factors of patients traced after being lost to follow-up from antiretroviral treatment in Lilongwe, Malawi. BMC Infect Dis.

[6] Geng EH, Bangsberg DR, Musinguzi N, Emenyonu N, Bwana MB, Yiannoutsos CT (2010). Understanding reasons for and outcomes of patients lost to follow-up in antiretroviral therapy programs in Africa through a sampling-based approach. J Acquir Immune Defic Syndr.

[7] Cardoso GP, Arruda A (2004). As representações sociais da soropositividade e sua relação com a observância terapêutica. Cien Saude Coletiva.

[8] Yuan Y, L'Italien G, Mukherjee J, Iloeje U (2006). Determinants of discontinuation of initial highly active antiretroviral therapy regimens in a US HIV-infected patient cohort. HIV Med.

[9] Zago AM, Morelato P, Endringer EA, Dan GF, Ribeiro EM, Miranda AE (2012). Abandonment of antiretroviral therapy among HIV-positive patients attended at the Reference Center for HIV/AIDS in Vitória, Brazil. J Int Assoc Physicians AIDS Care (Chic).

[10] Hughes AJ, Mattson CL, Scheer S, Beer L, Skarbinski J (2014). Discontinuation of antiretroviral therapy among adults receiving HIV care in the United States. J Acquir Immune Defic Syndr.

[11] Marega A, Pires P, Samuel J (2017). Antiretroviral treatments abandon determinants in HIV positive patients, Chiúre, Mozambique, 2015. Int J Res.

[12] Groh K, Audet CM, Baptista A, Sidat M, Vergara A, Vermund SH (2011). Barriers to antiretroviral therapy adherence in rural Mozambique. BMC Public Health.

[13] Micek MA, Gimbel-Sherr K, Baptista AJ, Matediana E, Montoya P, Pfeiffer J (2009). Loss to follow-up of adults in public HIV care systems in Central Mozambique: identifying obstacles to treatment. J Acquir Immune Defic Syndr.

[14] Kiguba R, Byakika-Tusiime J, Karamagi C, Ssali F, Mugyenyi P, Katabira E (2007). Discontinuation and modification of highly active antiretroviral therapy in HIV-infected Ugandans. J Acquir Immune Defic Syndr.

[15] Opio D, Semitala FC, Kakeeto A, Sendaula E, Okimat P, Nakafeero B (2019). Loss to follow-up and associated factors among adult people living with HIV at public health facilities in Wakiso district, Uganda: a retrospective cohort study. BMC Health Serv Res.

[16] Musheke M, Bond V, Merten S (2012). Individual and contextual factors influencing patient attrition from antiretroviral therapy care in an urban community of Lusaka, Zambia. J Int AIDS Soc.

[17] Schöni-Affolter F, Keiser O, Mwango A, Stringer J, Ledergerber B, Mulenga L (2011). Estimating loss to follow-up in HIV-infected patients on antiretroviral therapy: the effect of the competing risk of death in Zambia and Switzerland. PLoS One.

[18] Brasil, Ministério da Saúde, Secretaria de Vigilância em Saúde, Departamento de Doenças de Condições Crônicas e Infecções Sexualmente Transmissíveis (2022). Protocolo clínico e diretrizes terapêuticas para atenção integral às pessoas com infecções sexualmente transmissíveis - IST.

[19] Kalichman SC, Katner H, Banas E, Hill M, Kalichman MO (2020). HIV-related stigma and non-adherence to antiretroviral medications among people living with HIV in a rural setting. Soc Sci Med.

[20] Endalamaw A, Gilks CF, Ambaw F, Shiferaw WS, Assefa Y (2024). Explaining inequity in knowledge, attitude, and services related to HIV/AIDS: a systematic review. BMC Public Health.

[21] UNAIDS Índice de estigma em relação às pessoas vivendo com HIV/AIDS, Brasil: estudo revela como o estigma e a discriminação impactam pessoas vivendo com HIV e AIDS no Brasil.

[22] Masa R, Zimba M, Tamta M, Zimba G, Zulu G (2022). The association of perceived, internalized, and enacted HIV stigma with medication adherence, barriers to adherence, and mental health among young people living with HIV in Zambia. Stigma Health.

[23] Siqueira LR, Cunha GH, Galvão MT, Fontenele MS, Fechine FV, Medeiros MS (2022). Effect of lipodystrophy on self-esteem and adherence to antiretroviral therapy in people living with HIV. AIDS Care.

[24] Moçambique, Ministério da Saúde, Direcção Nacional de Saúde Pública (2023). Guião de cuidados do HIV do adulto, adolescente grávida, lactante e criança: guião de bolso.

[25] Brasil, Ministério da Saúde, Secretaria de Vigilância em Saúde, Departamento de DST e AIDS Nota técnica Nº 208/09-UAT/DST-AIDS/SVS/MS: orientações para abordagem consentida, alerta de má adesão aos antirretrovirais e critério de abandono ao tratamento.

[26] Page MJ, McKenzie JE, Bossuyt PM, Boutron I, Hoffmann TC, Mulrow CD (2021). The PRISMA 2020 statement: an updated guideline for reporting systematic reviews. BMJ.

[27] Bramer WM (2018). Reference checking for systematic reviews using Endnote. J Med Libr Assoc.

[28] Risk of Bias Tools RoB 2 tool: a revised Cochrane risk of bias tool for randomized trials.

[29] Vuylsteke B, Semdé G, Auld AF, Sabatier J, Kouakou J, Ettiègne-Traoré V (2015). Retention and risk factors for loss to follow-up of female and male sex workers on antiretroviral treatment in Ivory Coast. J Acquir Immune Defic Syndr.

[30] Agbaji OO, Abah IO, Falang KD, Ebonyi AO, Musa J, Ugoagwu P (2015). Treatment discontinuation in adult HIV-infected patients on first-line antiretroviral therapy in Nigeria. Curr HIV Res.

[31] Ibiloye O, Decroo T, Eyona N, Eze P, Agada P (2018). Characteristics and early clinical outcomes of key populations attending comprehensive community-based HIV care: experiences from Nasarawa State, Nigeria. PLoS One.

[32] Kiwanuka J, Waila JM, Kahungu MM, Kitonsa J, Kiwanuka N (2020). Determinants of loss to follow-up among HIV positive patients receiving antiretroviral therapy in a test and treat setting: a retrospective cohort study in Masaka, Uganda. PLoS One.

[33] Palombi L, Moda N (2021). Poverty, illiteracy, hunger and nomadism as social, cultural and economic reasons of treatment abandonment in HIV/AIDS patients in Mozambique. Biomed Prev.

[34] Ma J, Jin Y, Jiao K, Wang Y, Gao L, Li X (2023). Antiretroviral treatment interruption and resumption within 16 weeks among HIV-positive adults in Jinan, China: a retrospective cohort study. Front Public Health.

[35] Schilkowsky LB, Portela MC, Sá MC (2011). Factors associated with HIV/AIDS treatment dropouts in a special care unit in the City of Rio de Janeiro, RJ, Brazil. Rev Br Epidemiol.

[36] Shabalala FS, Vernooij E, Pell C, Simelane N, Masilela N, Spiegelman D (2018). Understanding reasons for discontinued antiretroviral treatment among clients in test and treat: a qualitative study in Swaziland. J Int AIDS Soc.

[37] Muga R, Egea JM, Sanvisens A, Arnal J, Tural C, Tor J (2004). Impact of Injecting drug use on the interruption of antiretroviral therapies. J Epidemiol Community Health.

[38] Robison LS, Westfall AO, Mugavero MJ, Kempf MC, Cole SR, Allison JJ (2008). Short-term discontinuation of HAART regimens more common in vulnerable patient populations. AIDS Res Hum Retroviruses.

[39] Tran DA, Ngo AD, Shakeshaft A, Wilson DP, Doran C, Zhang L (2013). Trends in and determinants of loss to follow up and early mortality in a rapid expansion of the antiretroviral treatment program in vietnam: findings from 13 outpatient clinics. PLoS One.

[40] Nshimirimana C, Ndayizeye A, Smekens T, Vuylsteke B (2022). Loss to follow-up of patients in HIV care in Burundi: a retrospective cohort study. Trop Med Int Health.

[41] Duff P, Kipp W, Wild TC, Rubaale T, Okech-Ojony J (2010). Barriers to accessing highly active antiretroviral therapy by HIV-positive women attending an antenatal clinic in a regional hospital in western Uganda. J Int AIDS Soc.

[42] Miller CM, Ketlhapile M, Rybasack-Smith H, Rosen S (2010). Why are antiretroviral treatment patients lost to follow-up? A qualitative study from South Africa. Trop Med Int Health.

[43] Nsoh M, Tshimwanga KE, Ngum BA, Mgasa A, Otieno MO, Moali B (2021). Predictors of antiretroviral therapy interruptions and factors influencing return to care at the Nkolndongo Health District, Cameroon. Afr Health Sci.

[44] Rodrigues M, Maksud I (2017). Abandono de tratamento: itinerários terapêuticos de pacientes com HIV/Aids. Saude Debate.

[45] Lanier ER, Ait-Khaled M, Scott J, Stone C, Melby T, Sturge G (2004). Antiviral efficacy of abacavir in antiretroviral therapy-experienced adults harbouring HIV-1 with specific patterns of resistance to nucleoside reverse transcriptase inhibitors. Antivir Ther.

[46] Kranzer K, Lewis JJ, Ford N, Zeinecker J, Orrell C, Lawn SD (2010). Treatment interruption in a primary care antiretroviral therapy program in South Africa: cohort analysis of trends and risk factors. J Acquir Immune Defic Syndr.

[47] Samji H, Taha TE, Moore D, Burchell AN, Cescon A, Cooper C (2015). Predictors of unstructured antiretroviral treatment interruption and resumption among positive individuals in Canada. HIV Med.

[48] Asad S, Hulgan T, Raffanti SP, Daugherty J, Wayne R, Sterling TR (2008). Sociodemographic factors predict early discontinuation of HIV non-nucleoside reverse transcriptase inhibitors and protease inhibitors. J Natl Med Assoc.

[49] McManus H, Petoumenos K, Brown K, Baker D, Russell D, Read T (2015). Loss to follow-up in the Australian HIV Observational Database. Antivir Ther.

[50] Seifu W, Ali W, Meresa B (2018). Predictors of loss to follow up among adult clients attending antiretroviral treatment at Karamara general hospital, Jigjiga town, Eastern Ethiopia, 2015: a retrospective cohort study. BMC Infect Dis.

[51] Modipane M, Khoza LB, Ingersoll K (2023). Barriers contributing to loss to follow-up among HIV-patients in Limpopo Province, South Africa: patients' and nurses' perspectives. Open Public Health J.

[52] Balogun M, Meloni ST, Igwilo UU, Roberts A, Okafor I, Sekoni A (2019). Status of HIV-infected patients classified as lost to follow up from a large antiretroviral program in southwest Nigeria. PLoS One.

[53] Assemie MA, Leshargie CT, Petrucka P (2019). Outcomes and factors affecting mortality and successful tracing among patients lost to follow-up from antiretroviral therapy in Pawi Hospital, Northwest Ethiopia. Trop Med Health.

[54] Pires PN, Marega A, Creagh JM (2017). Adesão à terapia antirretroviral em pacientes infetados pelo VIH nos cuidados de saúde primários em Nampula, Moçambique. Rev Port Med Geral Fam.

[55] Akilimali PZ, Musumari PM, Kashala-Abotnes E, Kayembe PK, Lepira FB, Mutombo PB (2017). Disclosure of HIV status and its impact on the loss in the follow-up of HIV-infected patients on potent anti-retroviral therapy programs in a (post-) conflict setting: a retrospective cohort study from Goma, Democratic Republic of Congo. PLoS One.

[56] Siti-Azrin AH, Norsaadah B, Lee SC, Suresh KC, Wan-Nor-Asyikeen WA (2023). Predictors of discontinuation of antiretroviral therapy among HIV-infected adults at Hospital Sungai Buloh: a 10-year retrospective cohort study. Gulhane Med J.

[57] Mbalinda SN, Bakeera-Kitaka S, Lusota DA, Musoke P, Nyashanu M, Kaye DK (2021). Transition to adult care: exploring factors associated with transition readiness among adolescents and young people in adolescent ART clinics in Uganda. PLoS One.

[58] SeyedAlinaghi S, Karimi A, Barzegary A, Pashaei Z, Zargari G, Kianzad S (2022). Prevalence and reasons of loss to follow-up in HIV clinics: a systematic review of current evidence. HIV AIDS Rev.

[59] Seong H, Choi Y, Kim M, Kim JH, Song JY, Kim SW (2023). Rate of and risk factors for loss to follow up in HIV-infected patients in Korea: the Korea HIV/AIDS Cohort Study. Infect Chemother.

